# Transcriptome investigation of anti‐inflammation and immuno‐regulation mechanism of taurochenodeoxycholic acid

**DOI:** 10.1186/s40360-021-00491-0

**Published:** 2021-04-29

**Authors:** Lige Bao, Dacheng Hao, Xu Wang, Xiuling He, Wei Mao, Peifeng Li

**Affiliations:** 1 College of Veterinary Medicine, Inner Mongolia Agricultural University, Hohhot, China; 2 Key Laboratory of Clinical Diagnosis and Treatment Techniques for Animal Disease, Ministry of Agriculture, Hohhot, China

**Keywords:** Taurochenodeoxycholic acid, RNA sequencing, Fibroblast‐like synoviocytes, Glucocorticoid receptor, Serine/arginine-rich splicing factor-9

## Abstract

**Background:**

Taurochenodeoxycholic acid (TCDCA) is one of the major active components in bile acid. It was proven to have inhibitory activities on inflammation and also participate in host immuno-regulation. TCDCA exerts anti-inflammatory and immuno-regulatory effects through the glucocorticoid receptor (GR) mediated genomic signaling pathway and the G protein-coupled bile acid receptor 5 (TGR5) mediated AC-cAMP-PKA signaling pathway. However, it is unclear whether GR or TGR5 plays an important role in the regulatory effects of TCDCA. In order to further investigate this effects mechanism of TCDCA, the research use the transcriptome to identify the major genes and pathway in the anti-inflammatory and immuno-regulatory effects.

**Methods:**

After the Fibroblast-like synoviocytes (FLS) being treated by different concentrations (10^− 5^, 10^− 6^ and 10^− 7^ M) of TCDCA for 12 h, the resulting mRNA was analyzed by RNA-seq. The differentially expressed genes were screened from sequencing results using bioinformatics techniques. In the next step, other published literature were referred in order to find out whether those genes mentioned above are related to inflammation. The final selected differentially expressed genes associated with inflammation were then validated by q-PCR and western blot assays.

**Results:**

Five genes associated with anti-inflammatory and immuno-regulatory effects, include Glyceraldehyde-3-phosphate dehydrogenase (GAPDH), Glutathione peroxidase 3 (GPX3), Serine/arginine-rich splicing factor-9 (SRSF9), Connective tissue growth factor (CTGF) and Cystatin B (CSTB) were identified. TCDCA at the concentrations of 10^− 5^, 10^− 6^ and 10^− 7^ M significantly (*p* < 0.05) up-regulate the mRNA and protein expression of SRSF9 and GPX3 and also up-regulate the mRNA expression of CSTB, CTGF and GAPDH. RNA-seq results of GPX3 and SRSF9 expression were consistent with q-PCR results, while q-PCR results of CTGF, GAPDH showed inconsistent with their RNA-seq results. Q-PCR result of CSTB expression also showed inconsistent with the RNA-seq result.

**Conclusions:**

The anti-inflammatory and immuno-regulatory activities of TCDCA are proven to be related to the up-regulation expression of GPX3, SRSF9 and CSTB.

## Background

**Fig. 1:**
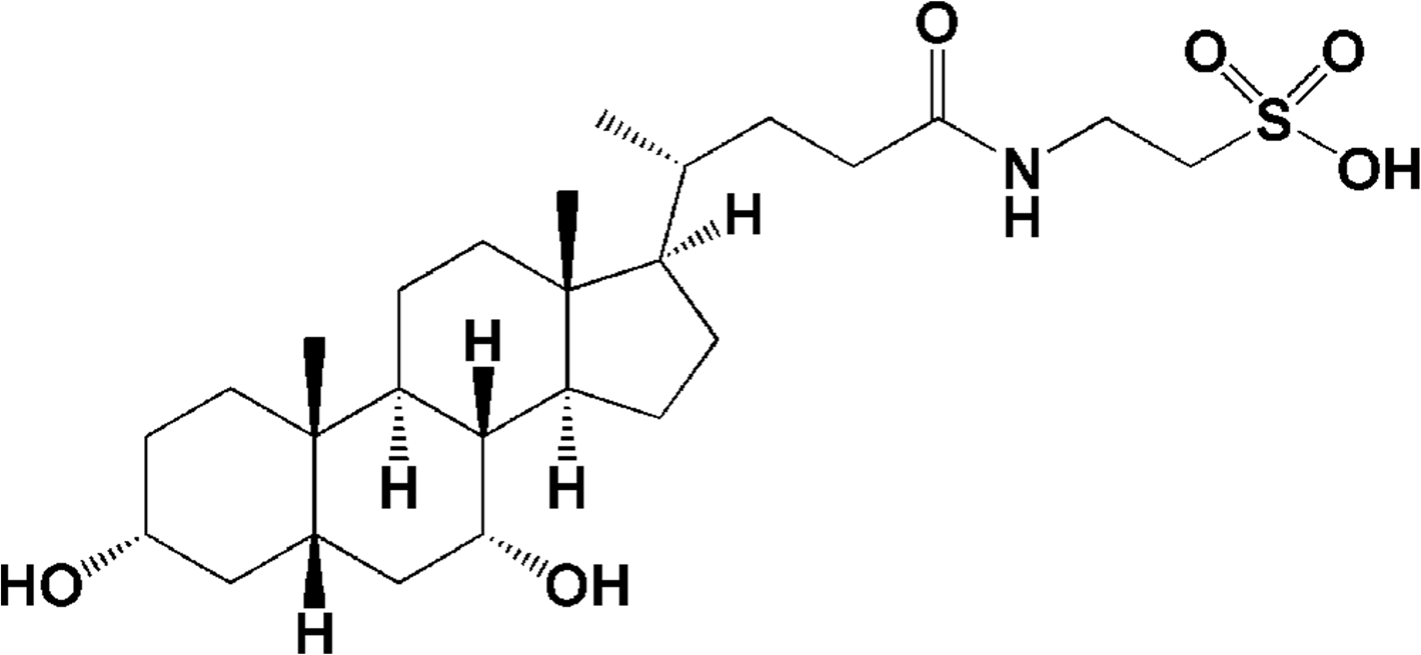
Chemical structure of TCDCA. Molecular weight: 499.69 Da, molecular formula: C_23_(OH)_2_H_37_CONHCH_2_CH_2_SO_3_H

Taurochenodeoxycholic acid (TCDCA) is one of the main active constituents of bile acids (BAs) [1], which is mainly found in bile from chickens, ducks, cattle, sheep, snakes, bear, and other species [2]. The IUPAC name is 3α,7α-dihydroxy-5β-24-cholanoyl-N-Taurine [1]. TCDCA is synthesized from taurine and chenodeoxycholic acid in the organisms (Fig. 1).

Our pervious research showed that TCDCA had inhibitory effects on both acute and chronic inflammation, and could also regulate immune response [3, 4]. TCDCA reduces capillary permeability and had anti-inflammatory activity against xylene induced mice earlobe edema [5]. It also inhibits toe swelling in complete Freund’s adjuvant (CFA) induced adjuvant arthritis in rats [5]. Studies have shown that TCDCA degrades the level of Leukotriene B_4_ (LTB4) in peripheral blood. The anti-inflammatory action of TCDCA attributed to down regulation of the expression of LTB_4_, NO and PEG_2_ gene. In addition, it inhibits the expression of TNF-α, IL-1β and IL-6 on both mRNA and protein level in synovial tissue and plasma of adjuvant arthritis (AA) rats [3, 6].

The immuno-regulatory activity of TCDCA had been reported, it increases the hemolysin, lysozyme and IgG levels in mouse serum, intensifies phagocytic function of mononuclear-peritoneal macrophages, enhances the percentage of CD_3_^+^, CD_4_^+^, and CD_19_^+^ cells and CD_4_^+^/CD_8_^+^ ratio in the peripheral blood of mice, and reduce the secretion of IL-1β in peritoneal macrophages [7, 8]. Additionally, TCDCA had regulatory effect on mitogen-stimulated mouse spleen lymphocyte immune function [9].

Other published studies demonstrated that TCDCA exerts anti-inflammatory and immuno-regulatory effects, affects the glucocorticoid receptor (GR) mediated genomic signaling pathway [10–12], as well as the G protein-coupled bile acid receptor 5 (TGR5) mediated AC-cAMP-PKA signaling pathway [13, 14]. However, comparison of the regulatory effects of TCDCA on GR and TGR5 had not yet been published, therefore, the objective of our study was to identify the major signaling pathway during the anti-inflammatory and immuno-regulatory effects of TCDCA using Fibroblast-like synoviocytes (FLS) recovered from AA rats’ model.

The development of molecular biology technologies has provided us a new technology termed RNA Sequencing (RNA-seq) for both mapping and quantifying transcriptome [15]. In order to further investigate the mechanism of anti-inflammatory and immuno-regulatory effects of TCDCA, and analyze the major genes and pathways involved, the transcriptional effects of TCDCA on FLS of AA rats was evaluated. Using RNA-seq could get new pathways, transcripts and key differentially expressed genes which come into effect in the process of anti-inflammation and immuno-regulation.

## Methods

### Adjuvant arthritis induction and cell culture preparation

The animal experiments were approved by the Animal Ethics Committee of Veterinary Medicine College, Inner Mongolia Agricultural University, Hohhot, China and conformed to national guidelines on the care and use of laboratory animals. Male Sprague Dawley (SD) rats (body weight = 100 ± 20 g), were obtained from Experimental Animal Center, Laboratory Animal Research Center, Inner Mongolia Medical University in China. All rats were housed in sawdust-lined cages at constant temperatures (22–25 ℃) and suitable humidity (40–70 %). Food and water were available *ad libitum*.

After one-week acclimation, AA rat model was established by injecting 0.1 mL CFA to 15 test animals in left hind paw pad intradermally. The mean body weight was recorded, and the day of model establishment was denoted as Day 0 [16]. Animals were fed with standard rodent chow, *ad libitum* for 13 to 15 days before euthanizing, by cervical dislocation following an inhalation anesthesia with isoflurane: 5 % for induction, 1–3 % for maintenance. Then, fresh synovial tissues were harvested. The tissue samples were washed three times with Dulbecco’s Phosphate Buffered Saline (DPBS), and cut into 1 ~ 2 mm^2^ tissue fragments then transferred to a culture flask. All flasks were placed inverted and incubated at 37 °C, in 5 % CO_2_ atmosphere for 2 h. Afterwards, fresh DMEM medium with 20 % fetal bovine serum was added, and incubated at 37 °C, in 5 % CO_2_ atmosphere for another 7 days. Synovial pieces were removed after the incubation; adherent cells were continuously cultured three generation in the same medium. After the confluence reached approximately 70 ~ 80 %, non-adherent cells were washed off with DPBS and adherent cells were harvested. The third-passage FLS in exponential growth phase were used for further experiments. After three passages, most cells comprise a homogeneous population of FLS [17].

### Total RNA extraction and study design

Fibroblast-like synoviocytes (FLS) were treated with TCDCA in different concentrations (10^− 5^, 10^− 6^ and 10^− 7^ M), while control FLS were incubated with DMEM alone (Table 1). All treated cells were incubated at 37 °C, in 5 % CO_2_ atmosphere for 12 h. Total RNA was extracted using Trizol reagent, quantity and integrity were assessed by 2100 Bioanalyzer (Agilent, USA). The RNA quality evaluation requests that the minimum amount of extracted RNA is 2 µg (concentration of ≥ 50 ng/µL). RIN (RNA integrity number) value for each test groups is required to be greater than 6.4. However, the value of 28 S/18S, whose ratio is a reasonable way to estimate RNA integrity, should be greater than 0.7 [18, 19].

**Table 1:** The treatment groups

Group	Treatment TCDCA concentration (M)
AA rat FLS group (FCA group)	-
AA rat FLS + TCDCA high dose treatment (AT10 group)	1 × 10^− 5^
AA rat FLS + TCDCA medium dose treatment (AT1 group)	1 × 10^− 6^
AA rat FLS + TCDCA low dose treatment (AT01 group)	1 × 10^− 7^

*AA* Adjuvant arthritis, *FLS* Fibroblast-like synoviocytes, *TCDCA* Taurochenodeoxycholic acid

### Transcriptome sequencing and cDNA library construction

Poly A containing mRNA was isolated from approximately 10 µg of total RNA using Poly-T oligo magnetic beads through hybridization, it was then purified and fragmented into small pieces using divalent cations under elevated temperature. The cleaved RNA fragments were reverse-transcribed to cDNAs and constructed the final cDNA library using the mRNA-Seq sample preparation kit protocol. The average insert size for the final cDNA library was 300 bp (± 50 bp). Then the paired-end sequencing was performed using Illumina Hiseq4000 (Illumina, USA).

### Sequencing data quality assessment and bioinformatics analysis

Using the Illumina paired-end RNA-seq method, the whole transcriptome was sequenced and generated a total of million paired-end reads of bp length. Before the re-assembly, the low-quality reads were removed. Reads from tested rat samples were aligned to the reference genome using HISAT2 (version 2.0.4), a portion of reads based on quality information were mapped to reference genome [20]. After the final transcriptome was generated, StringTie (version 1.2.4) and Ballgown (version 2.6.0) were used to estimate the expression levels of all transcripts. StringTie was used to evaluated the expression level for mRNAs by calculating Fragments Per Kilobase of exon model per Million mapped reads (FPKM) [21, 22]. The differentially expressed mRNAs and genes were analyzed [log2 (fold change) > 1, log2 (fold change) < -1; statistical significance (*p* < 0.05)] using Ballgown R package.

The raw data were processed, before obtaining any valid data. The ratio of bases with a mass value of ≥ 20 (Q20 %) and Q30 % are required exceed 90 %. At the same time, the ratio of GC content, was checked to determine whether there is a separation between AT and CG. The repeatability of each sample was determined based on the results of Principal Components Analysis (PCA) 3D map and the correlation analysis map. Expression level and differential expression level between different genes were further investigated if the result of repeatability was high. The differentially expressed genes in each sample group were evaluated by FPKM, P value (Probability value), gene with FPKM value greater than 100 or have major changing in FPKM value were chosen to be used as verification targets for further analysis [23]. Other function analyses used include GO (Gene Ontology) enrichment analysis and KEGG (Kyoto Encyclopedia of Genes and Genomes) signal pathway enrichment analysis, to select differentially expressed genes which were related to anti-inflammatory and immuno-regulatory effects.

After selecting the differentially expressed genes, Gene ID and/or the Transcript ID of those genes were searched in the protein annotation database (provided by LC Sciences). Name of the protein and its gene were determined based on the searching results, genes relating to anti-inflammation and immuno-regulation were identified based on others published literature.

### The RNA-seq profiles by quantitative real‐time PCR (q-PCR) assays

Differentially expressed genes related to anti-inflammatory and immuno-regulatory effects were selected for further validation by q-PCR analysis. Total RNA was extracted using the same batch of FLS as RNA-seq. The mRNA expression levels of five differentially expressed genes in both control and TCDCA (10^− 5^, 10^− 6^, 10^− 7^ M) treated groups were validated. The total mRNA was extracted from FLS in 6-well plates by AxyPrep Total RNA Extraction Kit. The ratio of OD_260/280_ and agarose gel electrophoresis were determined to ensure the quality of RNA [16]. Synthesis of cDNA was carried out using q-PCR (Hiscript^®^ Q RT SuperMix) following the manufacturer’s protocol. Total cDNA was used as starting material for quantitative real-time PCR [FastStart Universal SYBR Green Master equipped with Q7 system (ABI Biosystemss, USA)]. Q-PCR reaction system need total 20 µL of reaction mixture including 2 µL of cDNA, 10 µL of SYBR, 0.8 µL of specific target primers (10 µM)forward and reverse and 6.4 µL of double distilled H_2_O [17]. The q-PCR analysis was carried out using specific primers (Table 2). All data were calculated based on the comparative Ct formula and each sample was normalized by β-actin [24]. Relative mRNA expressions were analyzed according to the Ct values, based on the equation: 2^−ΔCt^ [ΔCt = Ct (SRSF9/GPX3/CSTB/CTGF/GAPDH) - Ct(β-actin)].

**Table 2:** Primers sequence

Gene name	Sequence (5′ → 3′)
SRSF9	Forward: ATTTGGTGTCATCCAGTTTCC
	Reverse: CAGATTTCCGAGTTCTTGTTTC
GPX3	Forward: GGTGTTGTACCAAACCGTGAG
	Reverse: ATCCCAGATTCCCTTCCTTAT
CSTB	Forward: GGAAAGAAACAAAATACAACG
	Reverse: TACCAGACCGACAAAGAAAAG
CTGF	Forward: AGTTCGTGTCCCTTACTCCCT
	Reverse: AAGACCTGTGCCTGCCATTAC
GAPDH	Forward: TCACCCCATTTGATGTTAGCG
	Reverse: GCAAGTTCAACGGCACAGTCA
β-actin	Forward: GGAGATTACTGCCCTGGCTCCTA
	Reverse: GACTCATCGTACTCCTGCTTGCTG

### Total protein extraction

FLS cells from the same batch used for the RNA-seq assay were used for total protein extraction. The protein expression level of differentially expressed genes in both control and TCDCA treated groups were verified. Extraction procedures followed the manufacturer’s protocol using M-PER Mammalian Protein Extraction Reagent kit. After the treatment, medium was removed and 1 mL of M-PER Reagent was added to each flask. All flasks were shaken gently for 5 min, then cells at the bottom of the flasks were scraped off. All cells were suspended in the reagent, vortexed and centrifuged at 14,000 g for 10 min at 4 °C. The supernatant was collected and stored at -80 ℃.

### Assessment of relationship between SRSF9 and GPX3 and inflammatory and immuno-regulatory process using western blotting

Protein standard curve was established using the Pierce™ BCA Protein Assay Kit and protein concentration in test samples were determined. For western blotting analysis, 6 µg of total protein per lane was separated by 12 % SDS-PAGE and blotted onto a PVDF membrane. Rabbit anti-mouse SRSF9 (1:500), goat anti-mouse GPX3 polyclonal antibodies (1:167) and β-actin mouse monoclonal antibody (1:3000) were used for protein detection. Proteins were then treated using secondary horseradish peroxidase (HRP)-conjugated rabbit anti-goat (1:3000), goat anti-mouse antibodies (1:3000), goat anti-rabbit antibody (1:3000) and Pierce SuperSignal West Femto chemiluminescent substrate (Thermo Fisher Scientific, USA). The bands were visualized by maximum sensitivity substrate. Chemiluminescence signals were measured using the ECL system (ImageQuantLAS4000, GE Healthcare). Protein signal intensity was measured by densitometric analysis using ImageJ software (version 1.48).

### Statistical analysis

All data were analyzed using SPSS 19.0 software, and presented as mean ± standard deviation (SD). T-test was used for two groups comparison, while one-way ANOVA was used when more than two groups were compared. *P* < 0.05 (represented as *), *p* < 0.01 (represented as **) and *p* < 0.001 (represented as ***) were considered as significant.

## Results

### The FLS cells

**Fig. 2:**
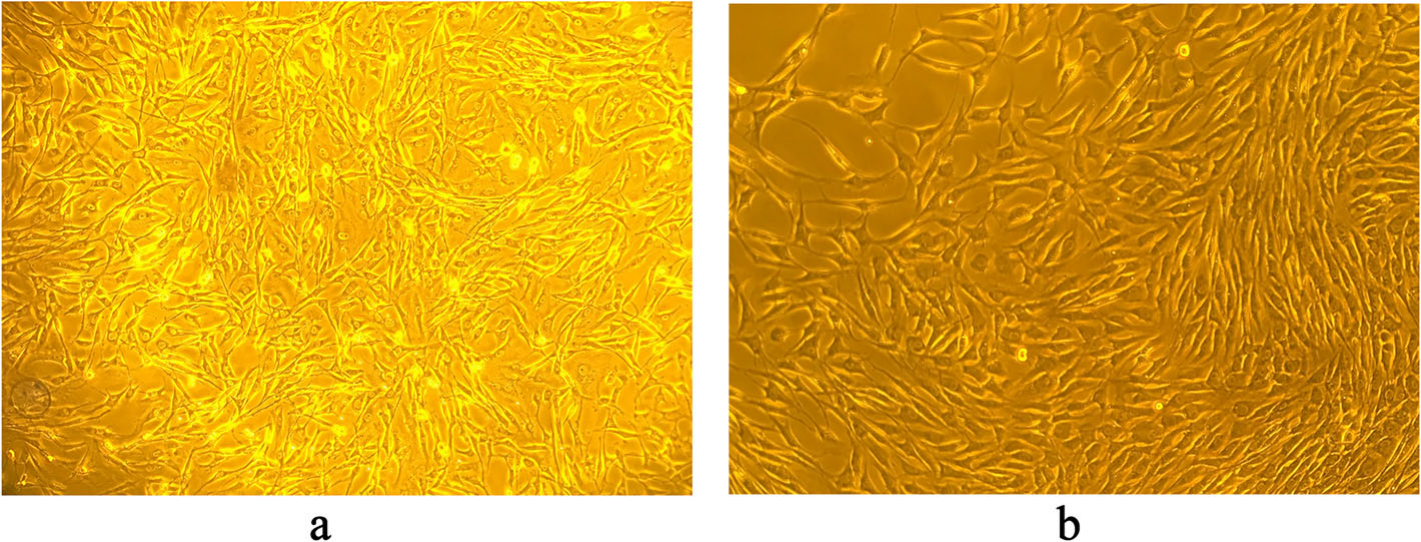
Fibroblast-like synoviocytes condition by light microscopy (200×)

FLS cell (Fig. 2a) was spindle shaped and had protruding protrusions. During the cell culture, when the confluence reached 50 ~ 60 %, it could be sub-cultured and the morphology of third generation cells were recorded (Fig. 2b). After the sub-culturing, cell changed into elongated shape, and dedifferentiate into fibroblast-like cells.

### Quality of total RNA and transcriptome sequencing quality control

The RNA quality shown that single signal peak of both 18 and 28 S were detected, which indicated the extracted RNA was purified and qualified, RIN values of all groups were greater than 6.4, and 28 S/18S > 0.7. Total extracted amount of RNA was beyond 2 µg, and concentration was measured up to 50 ng/µL, all parameters indicated the extracted RNA was qualified for transcriptome sequencing.

**Fig. 3:**
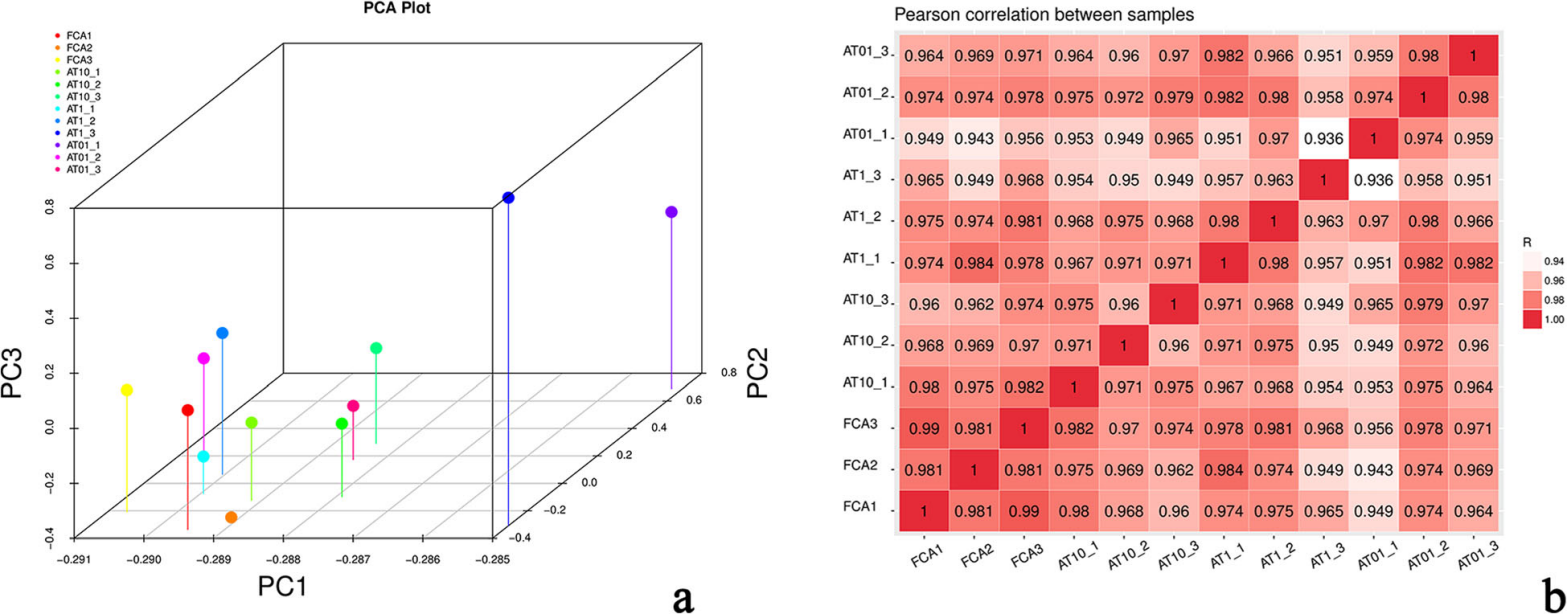
Three-dimensional image of PCA (**a**) and correlation cluster diagram (**b**)

Results from the comprehensive evaluations of the transcriptome sequencing quality are presented in Table 3. Data from 8 out of 12 samples were higher than 7G, while Q20 % and Q30 % of all samples were all higher than 90 %, and the proportion of GC content were all close to 50 % (Table 3). Results of repeatability analysis among samples were displayed in three-dimensional image of PCA (Fig. 3a), the distribution of sample peaks (10 out of 12) were close to each other. While correlation cluster diagram illustrated that sample correlation between each other was high and close to 1 (Fig. 3b).

**Table 3:** RNA-seq data quality analysis results

Sample name	Raw data		Valid data		Efficient(reads)%	Q20 %	Q30 %	GC content%
	Read	Base	Read	Base				
FCA1	54,383,990	8.16G	53,835,634	8.08G	98.99	99.58	95.01	51.50
FCA2	41,050,214	6.16G	40,649,424	6.10G	99.02	99.77	96.14	52.00
FCA3	40,700,236	6.11G	40,326,736	6.05G	99.08	99.76	95.82	51.50
AT10_1	40,741,106	6.11G	40,307,186	6.05G	98.93	99.60	95.16	51.00
AT10_2	57,742,958	8.66G	57,142,398	8.57G	98.96	99.42	92.66	50.50
AT10_3	51,520,986	7.73G	50,998,720	7.65G	98.99	99.62	93.96	50.50
AT1_1	54,593,420	8.19G	54,067,912	8.11G	99.04	99.60	93.56	51.00
AT1_2	46,452,368	6.97G	46,050,198	6.91G	99.13	99.63	93.87	51.50
AT1_3	50,198,580	7.53G	49,764,940	7.46G	99.14	99.32	91.97	51.00
AT01_1	56,340,820	8.45G	55,884,918	8.38G	99.19	99.38	92.94	52.50
AT01_2	52,741,640	7.91G	52,255,040	7.84G	99.08	99.58	94.17	51.50
AT01_3	85,115,910	12.77G	84,203,182	12.63G	98.93	99.58	94.00	50.50

In the experimental design, the AA rat FLS group was represented as FCA groups in the Table 1; AA rat FLS + TCDCA high dose treatment was represented as AT10 group; AA rat FLS + TCDCA medium dose treatment was represented as AT1 group; AA rat FLS + TCDCA low dose treatment was represented as AT01 group; The numbers 1, 2, and 3 represented three sets of repeated studies

### Differentially expressed genes analysis

**Fig. 4:**
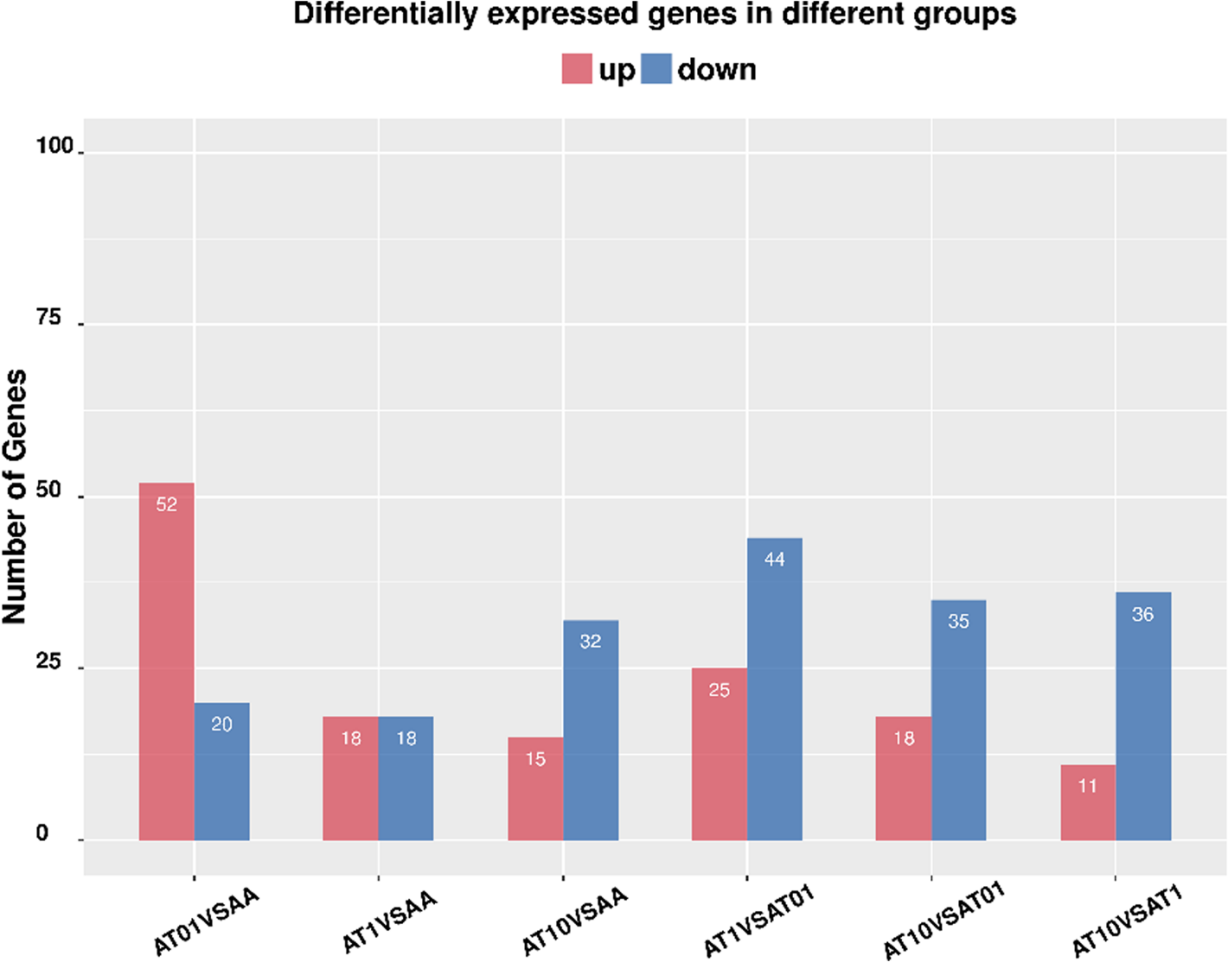
Differentially expressed gene regulation results among groups. **Red**: significantly up-regulated differentially expressed genes; **blue**: significantly down-regulated differentially expressed genes

**Fig. 5:**
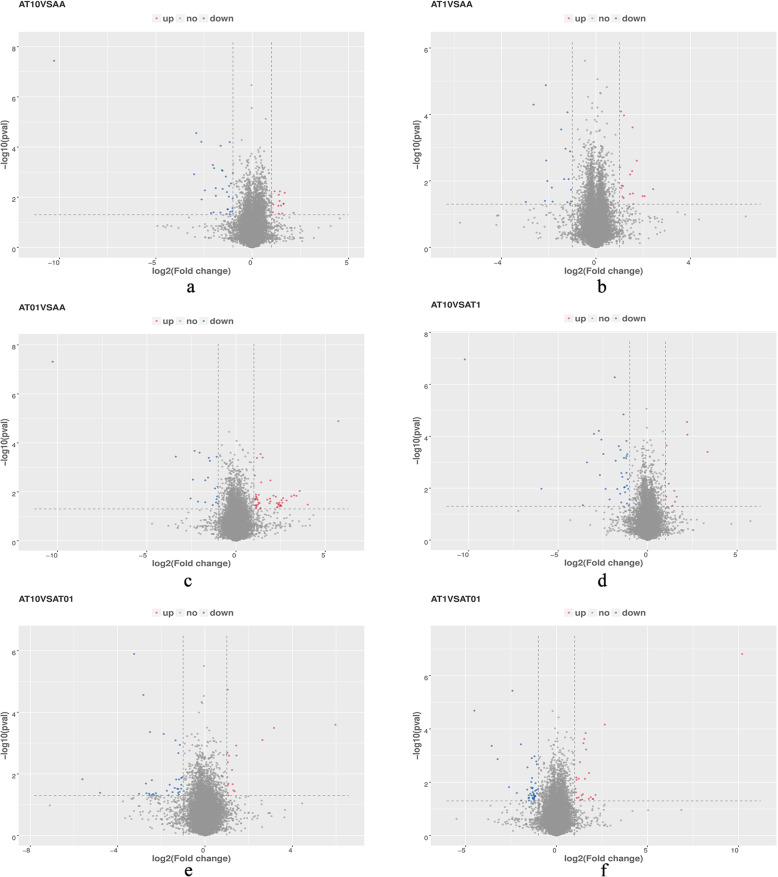
Volcano graph of 324 differentially expressed genes among groups. **Red**: significantly up-regulated differentially expressed genes; **blue**: significantly down-regulated differentially expressed genes; and **gray**: non-significantly differentially expressed genes

**Fig. 6:**
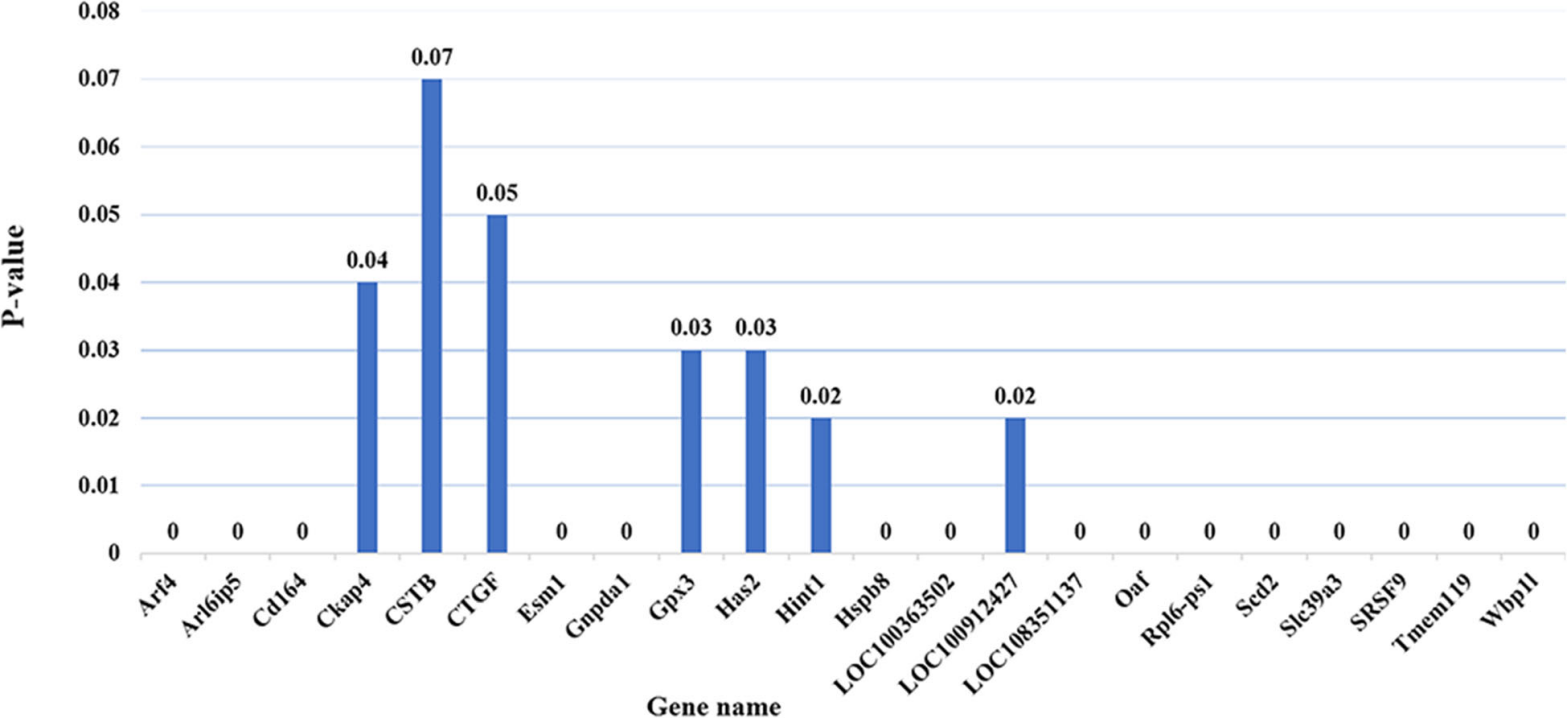
Differentially expressed genes in each group. *P*-value has been rounded to two digits

Differential analysis of the transcript expression from all samples were profiled. The results demonstrated that 324 genes were significantly altered (≥ 2-fold, *p* < 0.05), among them, 139 genes were upregulated and 185 genes were downregulated (Figs. 4 and 5). Further investigations on 324 differentially expressed genes were carried out, the specified differentially expressed genes in each sample group was then evaluated by FPKM and P value. Finally, 24 differentially expressed genes were selected between the groups and the results presented in Fig. 6.

### Anti‐inflammation and immuno‐regulation related differentially expressed genes

Five genes were identified to be related to anti-inflammation and immuno-regulation, which were GAPDH, GPX3, SRSF9, CTGF and CSTB (Table 4). Two other anti-inflammation related genes (Slc39a3 and LOC100363502) were also identified by referring to published literature. Slc39a3 (Zinc transporter ZIP3) is associated with anti-inflammatory effects, however, the expression level of it is low and not suitable for further analysis. Gene LOC100363502 and its translation product was identified as Cytochrome C, also known as CYC. It was reported that the increase of expression of CYC may relate to inflammatory activities. Since the subtype of CYC was not clear, no further analysis has been performed in this study.

**Table 4:** Anti-inflammatory and immune-regulatory effects related differentially expressed genes and their FPKM

No.	Gene ID	Gene Name	Protein Annotation	Group	FPKM					
					Before administration / High dose			After administration / Low dose		
1	MSTRG.13790	LOC108351137	Glyceraldehyde-3-phosphate dehydrogenase, GAPDH	AA VS AT10	1221.52	1284.88	1228.59	0	0	0
				AA VS AT01	1221.52	1284.88	1228.59	0	0	0
				AT10 VS AT1	0	0	0	1166.15	1257.69	1155.12
				AT1 VS AT01	1166.15	1257.69	1155.12	0	0	0
2	MSTRG.2701	GPX3	Glutathione peroxidase 3, GPX3	AA VS AA01	118.24	211.05	129.51	378.47	320.96	304.55
3	MSTRG.4849	SRSF9	Serine/arginine-rich splicing factor-9, SRSF9	AA VS AT1	14.19	15.7	15.56	33.01	35.53	30.19
4	MSTRG.121	CTGF	Connective tissue growth factor, CTGF	AA VS AT10	703.83	755.68	623.55	190.01	716.87	141.59
5	MSTRG.10440	CSTB	Cystatin B, CSTB	AA VS AT01	180.14	180.15	185.93	75.93	80.14	190.53
				AT1 VS AT01	187.17	187.48	174.88	75.93	80.14	190.53

Research indicated that other unselected differentially expressed genes were not likely to associate with inflammation, and few publications were found. So, only five of the identified differentially expressed genes were selected for further analysis.

### Protein and mRNA assessment results of SRSF9 and GPX3

**Fig. 7:**
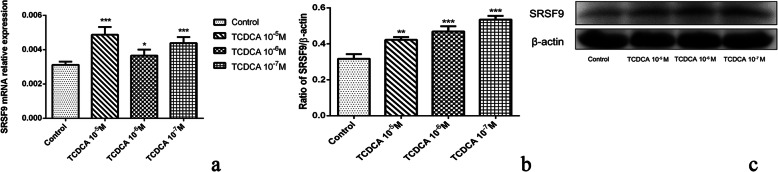
The mRNA (**a**) and protein (**b**) expression level of SRSF9. * *p* < 0.05; *** *p* < 0.01

**Fig. 8:**
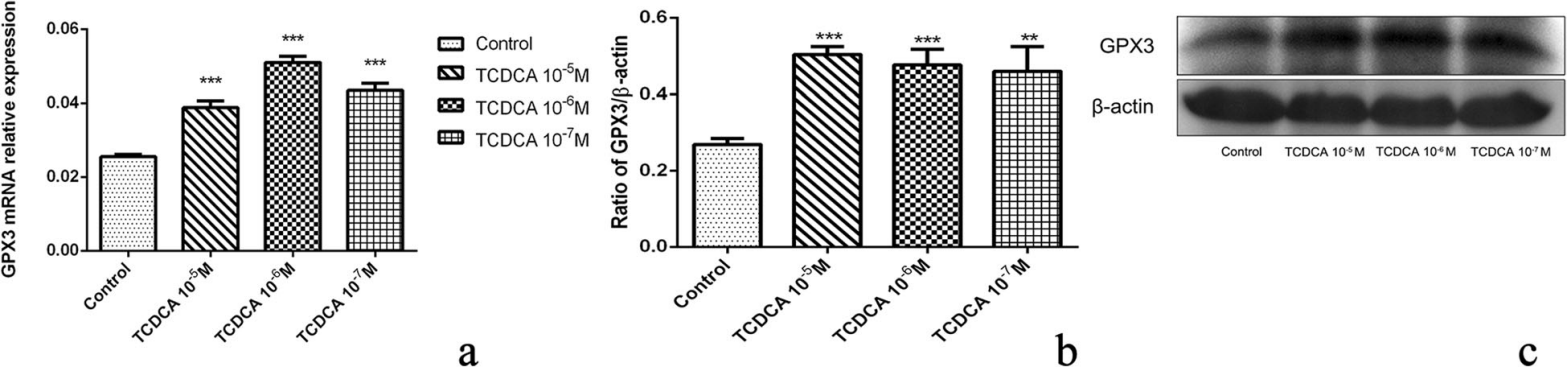
The mRNA (**a**) and protein (**b**) expression level of GPX3. * *p* < 0.05; *** *p* < 0.01

The correlation between gene SRSF9, GPX3 and anti-inflammatory and immuno-regulatory effects were verified by q-PCR and western blot assays. The mRNA level and protein expression levels of these two genes in control and TCDCA (10^− 5^ M, 10^− 6^ M, 10^− 7^ M) treated groups were analyzed. The results showed that mRNA and protein expression level of SRSF9 were significantly increased (Fig. 7) after treatment with TCDCA (10^− 5^ M, 10^− 6^ M, 10^− 7^ M), when compared to the control group (*p* < 0.05). TCDCA (10^− 5^ M, 10^− 6^ M, 10^− 7^ M) significantly (Fig. 8) enhanced the GPX3 mRNA and protein expression level, when compared with the control group (*p* < 0.05).

### mRNA assessment results of CSTB、CTGF and GAPDH

**Fig. 9:**
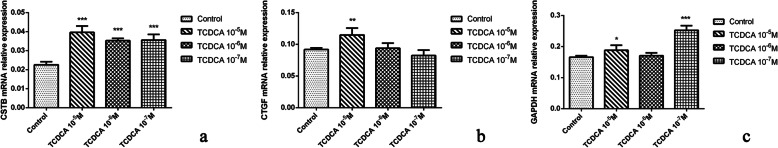
The mRNA expression levels of CSTB, CTGF and GAPDH. * *p* < 0.05; ** *p* < 0.01; *** *p* < 0.01

The mRNA expression levels of three selected genes (CSTB、CTGF and GAPDH) in both control and TCDCA treated groups were analyzed. The results illustrated that mRNA expression level of CSTB were significantly increased after treatment with TCDCA at different concentrations when compared to the mRNA level in control group (*p* < 0.05, Fig. 9a). While TCDCA (10^− 5^ M) treatment significantly (*p* < 0.05) enhance CTGF mRNA expression (Fig. 9b). The results showed that mRNA expression level of GAPDH were significantly increased (*p* < 0.05, Fig. 9c) after treatment with TCDCA (10^− 5^ M, 10^− 7^ M) compared to the control group.

## Discussion

TCDCA is synthesized with taurine and chenodeoxycholic acid in organism, and it is also one of the main bioactive substances of bile from animal bile. It has inhibitory effects on inflammation and could also regulate immune function [1, 3, 4]. The previous studies demonstrated that TCDCA exerts anti-inflammatory and immuno-regulatory effects, in both GR mediated genomic signaling pathway, and TGR5 mediated AC-cAMP-PKA signaling pathway [10–14]. In order to further investigate the major genes and pathways involved in the TCDCA relating to anti-inflammatory and immuno-regulatory effects, FLS from AA rats was used in our research, and transcriptome sequencing was used for differential gene investigation.

Differentially expressed genes were analyzed and selected by bioinformatics assays. Twenty-four differentially expressed genes were selected from RNA-seq results, among which five differentially expressed genes were identified by q-PCR. There were five genes relating to anti-inflammatory and immuno-regulatory effects, including SRSF9, GPX3, CTGF, CSTB and GAPDH.

Previous studies confirm that TCDCA can exert effects through GR [12, 25]. Then, the analytical of the SRSF9 showed that the mRNA expression and protein expression of SRSF9 were significantly increased in the treatment groups, this finding also confirmed our transcriptome sequencing results. According to Buoso et al. (2017), the up-regulated GR mRNA was caused by increasing expression of the splicing factor SRSF9 [26]. So TCDCA could increase the expression level of SRSF9 and enhanced the expression of GR as a result.

The results showed that the expression of GPX3 mRNA and protein was significantly increased in the TCDCA treatment groups. According to Manzanares’ finding (2008), patients with systemic inflammatory response syndrome (SIRS) showed significant decrease in the expression of GPX3 [27]. This indicates that GPX3 expression level will be significantly increased when inflammation response was inhibited. Therefore, the anti-inflammatory and immuno-regulatory effects of TCDCA could be related to the increased expression level of SRSF9, and are subsequently related to the increased expressions of GR and GPX3.

According to Maher’s findings (2014), the CSTB-deficient mice were significantly more sensitive to the lethal LPS-induced sepsis and could secrete higher amounts of pro-inflammatory cytokines IL-1β and IL-18 [28]. Q-PCR analytical results indicated that the CSTB mRNA expression levels of the TCDCA treatment groups were significantly increased, which is also consistent with some publications [28]. So, it is also opined that TCDCA can increase the expression level of CSTB.

The q-PCR results of differentially expressed genes CTGF and GAPDH were inconsistent with the transcriptome sequencing results. There was variation with transcriptome sequencing readouts, because Transcriptome sequencing and q-PCR are two different detection methods, and a certain degree of inconsistency (30 ~ 40 %) is both normal and reasonable phenomenon [29]. The final data and conclusions are subject to the q-PCR results. So, the inconsistent results were acceptable.

According to the above discussion, SRSF9 gene could modulate the expression of GR and have potential effect on GR modulated genomic signaling pathway. However, no differentially expressed genes were proven to have modulatory effects on the TGR5 mediated AC-cAMP-PKA signaling pathway. GR plays a critical role in the anti-inflammation and immune regulation after treatment of TCDCA in FLS model.

## Conclusions

In this research, five differentially expressed genes GAPDH, GPX3, SRSF9, CTGF and CSTB were selected and identified through the process and was investigated by bioinformatics analysis and assays. It could be concluded that the anti-inflammatory and immuno-regulatory effects of TCDCA is related to the activation of GR and the up-regulation of SRSF9, GPX3 and CSTB. This suggests that GR plays a more critical role in anti-inflammatory and immuno-regulatory effects than TGR5.

## Data Availability

The sequencing data determined in this work have been deposited in the NCBI GEO public database (https://www.ncbi.nlm.nih.gov/gds/) and are accessible through GEO series accession number GSE167513.

## Abbreviations

TCDCA: Taurochenodeoxycholic acid
CFA: Complete Freund’s adjuvant
GR: Glucocorticoid receptor
TGR5: G protein-coupled bile acid receptor 5
FLS: Fibroblast-like synoviocytes
AA: Adjuvant arthritis
RNA-seq: RNA sequencing
FPKM: Fragments per kilobase of exon model per million mapped reads
PCA: Principal components analysis
GO: Gene ontology
KEGG: Kyoto Encyclopedia of Genes and Genomes
UDCA: Ursodeoxycholic acid
GAPDH: Glyceraldehyde-3-phosphate dehydrogenase
GPX3: Glutathione peroxidase 3
SRSF9: Serine/arginine-rich splicing factor-9
CTGF: Connective tissue growth factor
CSTB: Cystatin B
